# Consequences of Gestational Malaria on Birth Weight: Finding the Best Timeframe for Intermittent Preventive Treatment Administration

**DOI:** 10.1371/journal.pone.0035342

**Published:** 2012-04-13

**Authors:** Bich-Tram Huynh, Nadine Fievet, Valérie Briand, Sophie Borgella, Achille Massougbodji, Philippe Deloron, Michel Cot

**Affiliations:** 1 UMR216, Institut de Recherche pour le Développement, Paris, France; 2 Université Paris Descartes, Paris, France; 3 UER de Parasitologie, Faculté des Sciences de la Santé, Cotonou, Bénin; University of Massachusetts Medical School, United States of America

## Abstract

To investigate the consequences of intermittent preventive treatment (IPTp) timing on birth weight, we pooled data from two studies conducted in Benin between 2005 and 2010: a prospective cohort of 1037 pregnant women and a randomised trial comparing sulfadoxine-pyrimethamine (SP) to mefloquine in 1601 women. A total of 1439 women (752 in the cohort and 687 in the SP arm of the randomised trial) who delivered live singletons were analysed. We showed that an early intake of the first SP dose (4 months of gestation) was associated with a lower risk of LBW compared to a late intake (6–7 months of gestation) (aOR = 0.5 p = 0.01). We also found a borderline increased risk of placental infection when the first SP dose was administered early in pregnancy (aOR = 1.7 p = 0.1). This study is the first to investigate the timing of SP administration during pregnancy. We clearly demonstrated that women who had an early intake of the first SP dose were less at risk of LBW compared to those who had a late intake. Pregnant women should be encouraged to attend antenatal visits early to get their first SP dose and a third dose of SP could be recommended to cover the whole duration of pregnancy and to avoid late infections of the placenta.

## Introduction

To prevent the consequences of malaria in pregnancy (MiP), intermittent preventive treatment (IPTp) strategy has been recommended since the early 2000s, and adopted by most African countries (including Benin in 2004). It consists in the administration under supervision of 2 curative doses of sulfadoxine pyrimethamine (SP) at least one month apart from the second trimester of pregnancy.

In Benin, a prospective cohort of pregnant women (“Strategy TO Prevent Pregnancy Associated Malaria” (STOPPAM)) was conducted from 2008 to 2010 to study immunological responses against MiP. In a preceding paper [1], we have highlighted the importance of the timing of malaria infections on the consequences of MiP. In early pregnancy, peripheral infections were associated with a higher risk of maternal anaemia at delivery, and with a decrease in the baby's mean birth weight. At the end of pregnancy, malaria infections increased the risk of maternal anaemia at delivery, without altering birth weight significantly. Hence the currently implemented schedule appears to leave the beginning of pregnancy unprotected, and the coverage of late pregnancy largely depends on the time of administration of the two doses of SP [2].

Therefore, as stressed by the World Health Organisation [3], there is a need to find an optimal timing for IPTp administration. To investigate this issue, the results from STOPPAM were compared, and then pooled with a randomised IPTp trial carried out in Ouidah, a nearby area, from 2005 to 2008.

## Materials and Methods

### Ethics statement

The 2 studies were approved by the ethics committees of the Research Institute for Development in France, and of the Science and Health Faculty in Benin. Written informed consent was given by all participants.

### Study area

**Figure 1:**
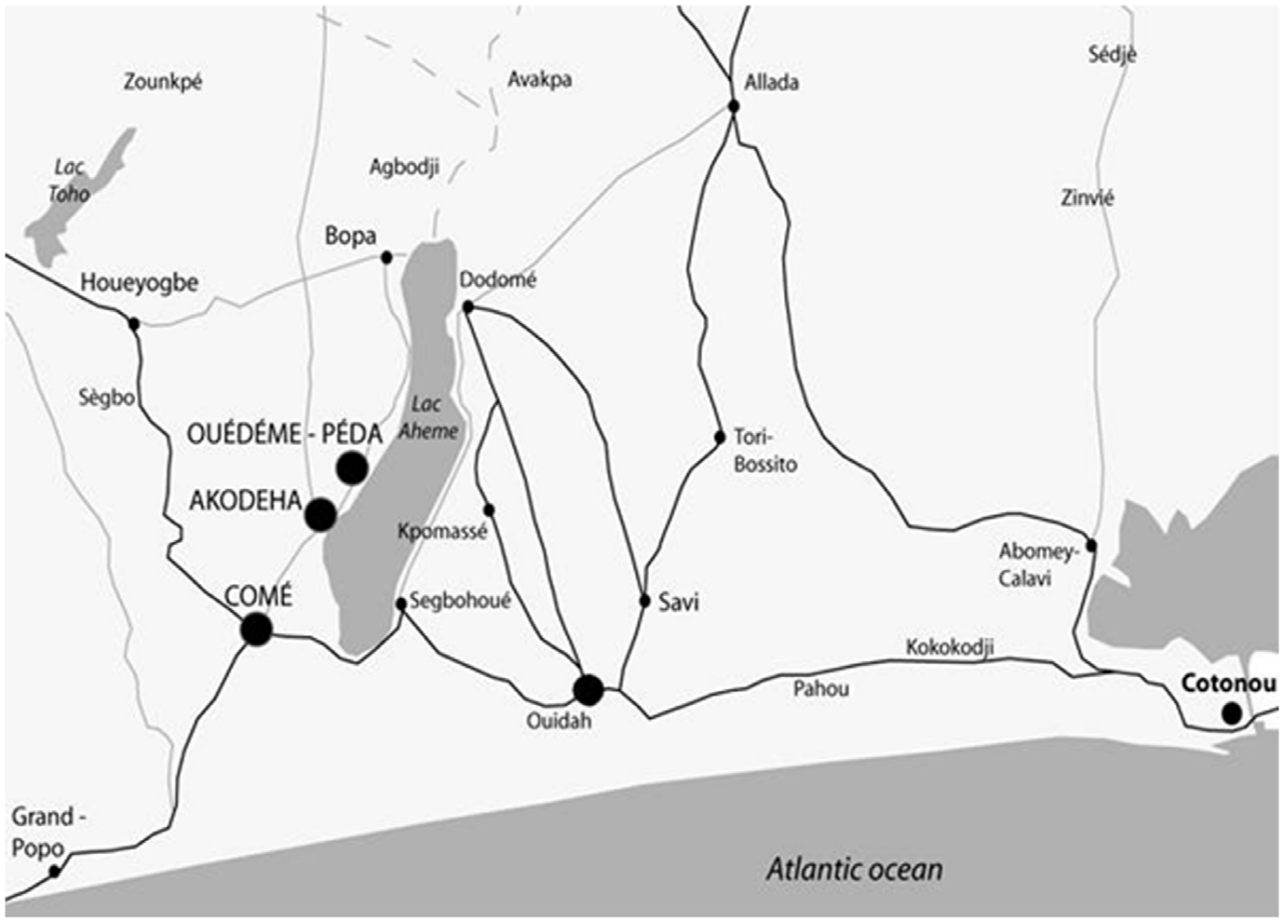
Study area of the randomized trial in Ouidah and STOPPAM.

The 2 studies have already been described in detail elsewhere [1], [4]. Briefly, the two sites are located in South Benin, 20 km apart from each other (figure 1). Ouidah area is semi-rural, whereas the area of STOPPAM study is more rural. Malaria transmission is high throughout the year, with two peaks from April to July and September to November. In a survey performed in 2005, in vivo resistance to SP was estimated at 50% in children under five in Ouidah [5]. In women followed in the trial, the prevalence of *pfdhfr* gene triple mutants (codons 51, 59, and 108) and *pfdhfr-pfdhps* quadruple mutants (*pfdhfr* codons 51, 59, and 108 and *pfdhps* codon 437) before first SP administration was 81% and 74%, respectively [6]. In STOPPAM study, the mutation prevalence remained stable over pregnancy with 85% quadruple and no quintuple mutants. (Tuikue Ndam, personal communication).

### Ouidah randomised trial

In Ouidah (40 km west from Cotonou), the controlled trial was conducted in 3 maternity clinics: Kindji, Kpassé and Hopital de Zone comparing SP (1,500–75 mg per dose) *versus* mefloquine (15 mg/kg per dose) for IPTp. Women under 28 weeks of gestation were included in the study. The first dose was scheduled between 16 and 28 weeks, and the second after 30 weeks of gestation, with at least 1 month in between. The two intakes were strictly supervised. Before each intake, a thick blood smear (TBS) was made. In case of clinical symptoms, women were encouraged to attend the clinic. At delivery, birth weight was measured and TBS, placental smears and blood samples were collected. The gestational age was assessed using the Ballard score [7], and performed by a single trained midwife within 72 hours after birth.

### STOPPAM study

In Comé area, a prospective cohort of pregnant women was followed in 3 maternity clinics: Comé central, Ouedeme Pedah and Akodeha. Women under 24 weeks of gestation were included in the study. Two doses of SP IPTp were administered following the national guidelines. Women were followed-up monthly from inclusion to delivery at the clinics. Like in Ouidah, women were encouraged to consult in case of symptoms. The gestational age was accurately estimated either by a midwife trained for ultrasound or by an obstetrician. On each visit (antenatal visit (ANV) or unscheduled visit), a rapid diagnostic test and a TBS were performed. At delivery, newborns were weighed. A TBS and placental smears were made and blood samples were drawn.

### Laboratory procedures

Placental smears from the two studies were stained with Giemsa. Malaria parasites were counted against 200 leukocytes. To ensure the quality of the smear reading, a control with a double reading was performed in both studies.

### Statistical analysis

Stata version 11 for Windows (Stata Corp, College Station, TX, USA) was used for all statistical analyses.

#### Outcome definitions

A birth weight under 2,500 g was considered as low birth weight. Placental infection was considered positive on the basis of the placental smears.

#### -Comparison of the 2 studies

To ensure comparability, we analyzed women only in the SP arm of the Ouidah trial. HIV infected women who received 3 doses of SP were excluded from the analysis. Only women with available dates for the two SP intakes, giving birth to a live singleton, were included in the analysis. We compared the baseline characteristics of the women, outcomes, and the timing of SP intakes between the 2 studies. Different outcomes were considered: LBW, birth weight, and placental infection. Differences in proportions and means were compared using the chi2 test (or Fisher's exact test) and the Student t-test, respectively.

#### Influence of the timing of SP administration on outcomes

In order to find the best timing for SP administration, we pooled the data from the 2 studies and performed a logistic or a linear regression depending on the outcome analyzed (binary or continuous).

We used 2 variables to account for the position and the duration of IPTp throughout the pregnancy for each woman. First, we defined a variable measuring the date of administration of first SP dose according to the gestational age estimated by the Ballard score or by ultrasound, depending on the study. It was categorised in 3 classes: 4 completed months of gestation (14 weeks of gestation and 6 days - 19 weeks), 5 months (19 weeks and 1 day- 23 weeks and 3 days), and 6–7 months of gestation (23 weeks and 4 days - 32 weeks). Then, we created a variable measuring the interval between the 2 SP intakes and transformed it into a four class variable corresponding to the quartiles.

Covariates were included in the initial models on the basis of the literature and on hypothesized underlying causal relationships (directed acyclic graphs (DAG)) [8], [9]. We considered the following covariates: sex of the newborn, parity (primigravidae or multigravidae), possession of bed net, education, mother's underweight (body mass index(BMI)<20 before pregnancy calculated by assuming a weight gain of 250 g per week of pregnancy), number of consultations (total number of ANVs and unscheduled visits, dichotomised: under or above the median), duration of pregnancy (3 classes: ≤38 weeks, 39 weeks and ≥40 weeks of gestation).

To evaluate the influence of the women's exposure to malaria, we created a four-level variable. The different levels correspond to the 4 possible SP administration patterns regarding the transmission periods: 2 doses during rainy season or dry season, first dose during rainy season and second during dry season, and first dose during dry season and second during rainy season. In the analysis, this variable was considered mainly as an indicator of both the time position and duration of the transmission period in the course of pregnancy. For instance, women who received 2 SP doses during the rainy season probably had a longer duration of pregnancy during the transmission period (and therefore had a longest duration to malaria exposure) compared to women who took 2 SP doses during dry season.

We identified each study (Ouidah trial or STOPPAM) as a covariate and included it in the initial model. Covariates were selected using a backward-stepwise strategy to obtain the final multivariable model, a p-value of <0.05 was considered statistically significant.

## Results

### Comparison of the 2 studies

In the Ouidah trial, 1,601 women were randomised: 802 in the mefloquine group and 799 in the SP group. In the SP arm, 733 women had 2 intakes of SP (4 were HIV infected; 62 received only one dose). Among them, we analyzed the data of 687 women with live singletons (10 sets of twins, 13 stillbirths, 1 miscarriage and 22 newborns with unavailable birth weights).

In the STOPPAM study, 982 women were followed-up and 845 had 2 intakes of SP (121 received a single dose or date of SP intake was unknown, 16 were HIV infected). After excluding 1 miscarriage, 27 stillbirths, 4 maternal deaths, 19 sets of twins and 42 newborns whose birth weight was unknown, 752 women and their live singletons were kept for analysis.

Finally, a total of 1,439 women and their live singletons were analyzed.

Table 1 presents the general characteristics of the mothers and the outcomes of the 2 studies.

**Table 1:** General characteristics of the women and outcomes of the 2 studies.

		Ouidah trial	STOPPAM	p value*
Number of women		687	752	
Mean age, (years)#		24.9 (5.5; 15–45)	26.4 (SD = 6.1, range = 15–45)	<0.001
Parity n (%)	Primigravidae	181 (18.2)	137 (26.4)	
	Multigravidae	506 (81.8)	615 (73.6)	<0.001
Education n (%)	No education	307 (44.7)	425 (56.5)	
	Partial or complete primary	259 (37.7)	212 (28.2)	<0.001
	Secondary	121 (17.6)	115 (15.3)	
Weight before pregnancy n (%)	BMI†≥20	491 (72.1)	474 (63.8)	
	BMI<20	190 (27.9)	269 (36.2)	0.001
Bed net at enrollment n (%)	No	209 (30.4)	511 (68.1)	
	Yes	478 (69.6)	239 (31.9)	<0.001
Total number of visits n (%)	≥5 visits	300 (43.7)	593 (78.9)	
	<5 visits	387 (56.3)	159 (21.1)	<0.001
Newborn sex n (%)	Male	349 (50.9)	387 (51.5)	
	Female	336 (49.1)	365 (48.5)	0.85
Low birth weight n (%)	No	628 (91.4)	672 (89.4)	
	Yes	59 (8.6)	80 (10.6)	0.19
Mean weight at delivery (g)#		3037.1 (429.6; 1350–4434)	3008.23 (SD = 474.1; range = 1250–4850)	0.23
Placental infection n (%)	No	583 (95.9)	579 (88.7)	
	Yes	25 (4.1)	74 (11.3)	<0.001
Interval time between the last menstruation period and the first intake, days (SD)		159.6 (23.3)	144.7 (21.5)	<0.001
First SP dose intake timing n (%)	6–7 months of gestation	287 (41.8)	120 (15.9)	
	5 months of gestation	277 (40.3)	357 (47.5)	
	4 months of gestation	123 (17.9)	275 (36.6)	
Interval time between the 2 doses, days (SD)		60.3 (23.1)	35.4 (9.9)	<0.001

# mean, (Standard deviation (SD); range).

* Differences between the 2 studies in proportions and means were compared using the χ^2^ and the Student t-test, respectively.

† Body mass index.

In the STOPPAM study, there were more primigravidae than in Ouidah (26.4% vs 18.2%), while the women were older on average (26.4 years vs 24.9). More women received no education in the STOPPAM study (56.5% vs 44.7%). In both studies, women were from close ethnic groups of South West Benin. More women declared they owned a bed net at enrolment in the Ouidah trial than in the STOPPAM study (69.6% vs 31.9%, p<0.001). There were more women in the STOPPAM study who attended more than the median 5 visits during their follow-up (78.9% vs 43.7%; p<0.001).

The first dose of IPTp was given later in the Ouidah trial than in the STOPPAM study (159.6 days (standard deviation (SD) = 23.3) (22 weeks and 5 days of gestation) vs 144.7 (SD = 21.5) (20 weeks and 4 days of gestation); p<0.001). The interval between the 2 doses was longer in Ouidah compared to the STOPPAM study (60.3 (SD = 23.1) vs 35.4 days (SD = 9.9) p<0.001). The mean birth weight and proportion of LBW were 3,008.2 g (SD = 474.1) and 10.6% in the STOPPAM study and 3,037.1 g (SD = 429.6) and 8.6% in the Ouidah trial, respectively. Neither mean birth weight nor LBW differed significantly between the 2 studies. Among the 1439 analyzed women, information on placental infection was missing for 178 women. However, age, education, use of bed net, proportion of underweight women were the same in women with available outcomes and those without (data not shown). The proportion of placental infection was higher in the STOPPAM study than in the Ouidah controlled trial (11.3% vs 4.1%; p<0.001).

### Influence of SP timing on birth weight and placental infection

After pooling the two studies, multivariable logistic regression showed that LBW was associated with a 0.5 decreased risk (p = 0.01) when the first dose was given early during pregnancy (4 months of gestation), compared to a late intake (6–7 months of gestation). There was no relation between the risk of LBW and the interval between doses. The risk of LBW was significantly higher for primigravidae and low-BMI women than for multigravidae and normal-BMI (aOR = 2.4; p<0.001 and aOR = 1.7; p = 0.009, respectively). The administration of 2 SP doses during the dry season *versus* rainy season resulted in a lower risk of LBW. As expected, women with a shorter duration of pregnancy had an increased risk of LBW (table 2).

**Table 2:** Factors significantly associated with low birth weight (Multivariate analysis performed on 1439 women and adjusted on newborn's gender, study, number of consultations, bed net and education).

		Adjusted OR*	95% Confidence interval	p value
First dose intake	6–7 months of gestation	1		
	5 months of gestation	0.8	[0.5, 1.3]	0.46
	4 months of gestation	0.5	[0.3, 0.9]	0.01
Interval time between the 2 doses	≤31 days	1		
	32–37 days	0.9	[0.5, 1.5]	0.65
	38–61 days	0.7	[0.4, 1.3]	0.3
	>62 days	1.0	[0.6, 1.9]	0.8
Parity	Multigravidae	1		
	Primigravidae	2.4	[1.6, 3.6]	<0.001
Underweight	BMI†≥20	1		
	BMI<20	1.7	[1.1, 2.5]	0.009
Duration of pregnancy	≥40 weeks of gestation	1		
	39 weeks of gestation	2.0	[1.1, 3.7]	0.02
	≤38 weeks of gestation	10.7	[6.2, 18.5]	<0.001
Transmission period	Dose 1 and dose 2 during rainy season	1		
	Dose 1 during rainy season and dose 2 during dry season	0.8	[0.4,1.5]	0.50
	Dose 1 during dry season and dose 2 during rainy season	0.8	[0.4,1.4]	0.4
	Dose 1 and dose 2 during dry season	0.6	[0.3,0.9]	0.05

* OR, odds ratio;

† Body mass index.

A first SP dose administered at the fourth or fifth month of gestation was associated to an increase in mean birth weight (138.2 g, p<0.001, and 72.5 g; p = 0.008, respectively) compared to a late intake (6–7 months of gestation). Similarly to LBW, no association was observed between the time between SP doses and birth weight. Mean birth weight was higher in the Ouidah trial than in the STOPPAM study (an increase of 63.1 g; p = 0.04). Having received 2 SP doses during the dry season resulted in an increase of mean birth weight (73.1 g; p = 0.02) compared to 2 doses during the rainy season. Low BMI, first pregnancy, foetal gender, low number of consultations and short duration of pregnancy were also associated with a decrease in mean birth weight (table 3).

**Table 3:** Factors significantly associated with mean birth weight (Multivariate analysis performed on 1439 women and adjusted on bed net, education).

		Adjusted difference in mean birth weight (g)	95% Confidence interval	p
First dose intake	6–7 months of gestation	0		
	5 months of gestation	72.5	[18.7, 126.4]	0.008
	4 months of gestation	138.2	[75.25 200.9]	<0.001
Interval time between the 2 doses	≤31 days	0		
	32–37 days	12.0	[−47.0, 71.1]	0.69
	38–61 days	3.2	[−59.8, 66.3]	0.98
	>62 days	−33.1	[−107.9, 41.7]	0.36
Parity	Multigravidae	0		
	Primigravidae	−179.7	[−230.6, −128.8]	<0.001
Study	STOPPAM	0		
	Ouidah trial	63.1	[1.6, 124.6]	0.04
Underweight	BMI*≥20	0		
	BMI<20	−132.5	[−178.0, −87.0]	<0.001
Duration of pregnancy	≥40 weeks of gestation	0		
	39 weeks of gestation	−147.9	[−196.8, −99.0]	<0.001
	≤38 weeks of gestation	−422.4	[−477.3, −367.5]	<0.001
Newborn's gender	Male	0		
	Female	−100.4	[−142.1, −58.7]	<0.001
Number of consultations	≥5 consultations	0		
	<5 consultations	−66.1	[−110.0, −22.1]	0.03
Transmission period	Dose 1 and dose 2 during rainy season	0		
	Dose 1 during rainy season and dose 2 during dry season	26.5	[−43.9, 96.9]	0.46
	Dose 1 during dry season and dose 2 during rainy season	44.3	[−25.9, 114.5]	0.22
	Dose 1 and dose 2 during dry season	73.1	[10.6, 135.6]	0.02

* Body mass index.

Although not significant, we observed a marginal increase in the risk of placental infection with an early first SP dose (aOR = 1.7; p<0.1). No association between placental infection and the interval between doses was observed. Mothers from the Ouidah trial had a reduced risk for a placental infection (aOR = 0.4; p = 0.005) and primigravidae presented with a greater risk of placental infection (aOR = 2.6; p<0.001) (table 4).

**Table 4:** Factors significantly associated with placental infection (Multivariate analysis performed on 1261 women and adjusted on newborn's gender, number of consultations, bed net, underweight, education, duration of pregnancy and transmission period).

		Adjusted OR*	95% Confidence interval	p value
First dose intake	6–7 months of gestation	1		
	5 months of gestation	1.2	[0.6, 2.1]	0.6
	4 months of gestation	1.7	[0.9, 3.1]	0.1
Interval time between the 2 doses	≤31 days	1		
	32–37 days	1.0	[0.6, 1.8]	0.84
	38–61 days	1.4	[0.8, 2.5]	0.26
	>62 days	0.7	[0.3, 1.7]	0.43
Parity	Multigravidae	1		
	Primigravidae	2.6	[1.6, 4.1]	<0.001
Study	STOPPAM	1		
	Ouidah trial	0.4	[0.2, 0.8]	0.005

* OR, odds ratio.

## Discussion

We investigated the importance of the timing of SP IPTp by analysing 2 follow-ups of women throughout pregnancy in Benin. We showed that the moment of administration of the first dose of SP had a significant influence on birth weight. An early intake of the first SP dose was associated with a lower risk of LBW compared to a late intake (a reduction by half) and with an increase in mean birth weight (more than 100 g). These findings are consistent with what we found in a preceding paper [1] and with the results of Cottrell et al. [10]. These studies showed that malaria infections during early pregnancy (<19 weeks) were particularly harmful for the mother and the newborn.

The timing of SP administration was only marginally associated with placental infection. The fact that the rate of placental infection was lower in the Ouidah trial than in the STOPPAM study may be explained by a later administration of the second SP dose, as the infection of the placenta at delivery probably reflects a late peripheral infection. An alternative explanation involves a better compliance due to the strict supervision of SP administration according to the directly observed therapy scheme in the randomised trial.

The timing of IPTp thus seems to act differently on LBW and placental infection. Foetal growth is maximal from the middle of the second trimester until the last month of pregnancy [11], therefore an early intake of SP may clear placental infections before the maximal growth velocity, allowing the foetus to grow harmoniously. On the contrary, a late intake susceptible to clear the placenta at delivery, may not improve in the same extent the foetal growth. A recent review highlighted different mechanisms by which malaria infections could lead to foetal growth restriction and therefore LBW [12]. Two periods appear to be crucial. Early infections may alter the angiogenesis causing placental insufficiency, whereas later infections may contribute to placental dysfunction through inflammation of the syncytiotrophoblast, decreasing nutrient transport or causing hormone dysregulation. Consequently, an early IPTp intake, by allowing a correct process of placentation, may well protect from LBW and not from placental infection, which occurs later in the course of pregnancy.

Although the ideal way to evaluate the best time to start IPTp is a randomized trial comparing different timings, raising appropriate funding for such a trial, not involving new antimalarial drugs, would not be easy. In Benin, two investigations on pregnant women receiving SP-IPTp were available, thus offering a unique opportunity to answer this question without building up a complete trial. In the STOPPAM study, women were encouraged to attend ANVs early, and they were offered ultrasound for the assessment of gestational age, which may have motivated them for early attendance at the clinic. No such facilities were proposed in the Ouidah trial, hence on average the first ANV was performed later than in STOPPAM.

The women differed regarding the study they originated from. STOPPAM women were older and had a lower school attendance compared to those from Ouidah. There were also more underweight individuals and primigravidae in STOPPAM, probably because of the more rural setting of this study. Getting free of charge follow-up, STOPPAM women's attendance to visits was higher, but they were less likely to possess an insecticide-treated net at inclusion than in Ouidah. However, the two studies were close to each other in terms of space and time. In both studies, the same type of population (pregnant women) was followed, under comparable transmission conditions and the same prevention strategy against MiP (SP IPTp). Finally, because we adjusted for all possible confounding factors in the different statistical models, it seems unlikely that pooling the two datasets could have led us to seriously biased results.

We could also verify the usual associations of known risk factors, such as a shorter duration of pregnancy, foetal sex, and maternal underweight with LBW, in agreement with previous studies on MiP [13], [14]. We assume that the finding of a lower rate of LBW associated with the intake of 2 SP doses during the dry season, compared to the rainy season, reflects the deleterious effect of the transmission season (and the associated increase in exposure to mosquito infective bites during rainy season) on pregnancy outcomes, rather than a higher efficacy of IPTp in this particular period.

Finally, we compared baseline characteristics of the women, such as education level or nutritional status assessed by the BMI, with the gestational age at enrolment, to rule out a potential selection bias (data not shown). We did not evidence any difference in the nutritional status, while a higher level of education was found in women who enrolled late in pregnancy (≥4 months, compared with <4 months), thus confirming the relation between the timing of IPTp and low birth weight.

It is noticeable that in the analyses of the factors associated with mean birth weight or placental infection, the Ouidah trial was associated to an overall higher protection than the STOPPAM study, even considering the later administration of IPTp in the former. In Ouidah trial, SP was administered under direct supervision whereas the supervision was looser in the STOPPAM study. These results are consistent with the findings of another study in a nearby site, Tori Bossito [15], where IPTp was given by the national health system. In this study, rates of placental infection and LBW were quasi-identical to those found in STOPPAM. The high degree of protection achieved by the randomized trial may logically be related with the strict respect of the directly observed therapy scheme.

The estimation of gestational age differed in the 2 studies, Ballard score in Ouidah and ultrasound scan in STOPPAM. In the Ouidah trial, all Ballard scores were measured exclusively by one specifically trained midwife to avoid inter-operator variability. All postnatal examinations were made within 72 hours (at a median time of 14 hours). In the STOPPAM study, ultrasound scan was precociously performed by only 2 operators. Therefore, the assessment of gestational age was made carefully in both studies and could not lead to biases. Moreover, we made the same analysis for each study separately, and found significant results for the different outcomes (data not shown). By combining the 2 studies, we confirmed the results while increasing the power of the analysis.

SP IPTp has already demonstrated its efficacy in West Africa [16], [17], [18], [19] as well as in East Africa [20], [21], in reducing the risk of LBW. By delivering the first SP dose earlier during the second trimester of pregnancy, we observed a gain of 100 g in mean birth weight, which is an important step comparable to the improvement (110 to 170 g) previously achieved by the shift from prophylaxis, either SP or chloroquine, to IPTp [16], [22]. Women should then be encouraged to attend antenatal visits early, as soon as they feel the quickening (15–20 weeks of gestation) to get their first dose of SP. This measure should not disrupt existing guidelines and minor efforts, regarding the considered benefit, will be required for a successful change.

A possible drawback of an early intake of SP IPTp is the lower protection during late pregnancy. Indeed, in the STOPPAM study, two thirds of the infected symptomatic women during unscheduled visits were found in late pregnancy, after the sixth month. [23]. Similar observations were made in Ouidah (V. Briand, personal communication): women were seen at unscheduled symptomatic visits between 6 and 8 weeks after IPTp intake on average. From a pathophysiological point of view, a recent article [24] suggested that using SP for IPTp could induce a competitive facilitation of the most highly resistant parasites, responsible for higher levels of parasitaemia and possibly more acute symptoms. To avoid such an inconvenience, a third dose should be considered at the end of pregnancy. A few studies have investigated the efficacy of more than 2 SP doses in HIV negative women, suggesting a better efficacy of these schemes [25], [26], providing a better coverage of pregnancy as well as a better efficacy to overcome increasing resistance [3]. A very recently published randomised trial in Mali [27] has shown the superiority of a three-dose over two-dose SP IPTp on both placental infection and LBW. In sub-Saharan Africa, the attendance to ANVs is high: 70% of the women attend at least once and among them, 95% attend twice and more than half attend 4 times [11]. The World Health Organisation recommends 4 ANVs including 3 after quickening for a good pregnancy surveillance through the “focused antenatal care”. A 3 dose-IPTp after the quickening could then be entirely part of the focused antenatal care strategy.

In conclusion, our study is to our knowledge the first to investigate the timing of SP administration during pregnancy. The results clearly show that women who had an early intake of the first dose of SP were less at risk of LBW compared to those with a later intake. In the context of increasing resistance to SP, researches are focusing on the finding of new candidates to replace SP for IPTp, if possible safe during the first trimester of pregnancy. It is of the utmost importance to ensure that future trials take into account the timing of the administration of drugs, and particularly an early intake and the full coverage of the pregnancy period.
